# Major Bioactive Compounds in Essential Oils Extracted From the Rhizomes of *Zingiber zerumbet (L) Smith*: A Mini-Review on the Anti-allergic and Immunomodulatory Properties

**DOI:** 10.3389/fphar.2018.00652

**Published:** 2018-06-20

**Authors:** Ji Wei Tan, Daud Ahmad Israf, Chau Ling Tham

**Affiliations:** ^1^Department of Biomedical Science, Faculty of Medicine and Health Sciences, Universiti Putra Malaysia, Seri Kembangan, Malaysia; ^2^School of Science, Monash University Malaysia, Bandar Sunway, Malaysia

**Keywords:** *Zingiber zerumbet*, rhizome, essential oil, anti-allergic, immunomodulatory, mini-review

## Abstract

*Zingiber zerumbet (L) Smith* is part of the *Zingiberaceae* family, one of the largest families of the plant kingdom. *Z. zerumbet* is a perennial, aromatic and tuberose plant that grows in humid locations where its center of distribution is located in the South-East Asia region. This plant has been traditionally used in foods and beverages and for ornamental purposes. Although many studies have reported on the biomedical applications of *Z. zerumbet*, the anti-allergic effects of *Z. zerumbet* and its major bioactive compounds have not yet been summarized in detail. Many major metabolites that have been reported to contain anti-allergic properties are terpene compounds which can be found in the essential oil extracted from the rhizomes of *Z. zerumbet*, such as zerumbone, limonene, and humulene. The rhizome is among the part of *Z. zerumbet* that has been widely used for many studies due to its exceptional biomedical applications. Most of these studies have shown that the essential oil, which can be obtained through hydro-distillation of the rhizomes from *Z. zerumbet*, is enriched with various active metabolites. Therefore, this mini-review provides an overview of the main aspects related to the anti-allergic and immunomodulatory properties of the major bioactive compounds found in the essential oils extracted from the rhizomes of *Z. zerumbet*, with the aim of demonstrating the importance of essential oil extracted from the rhizomes of *Z. zerumbet* and its bioactive compounds in the treatment of allergy and allergy-related diseases, in addition to other widely reported and extensively studied biomedical applications.

## Introduction

*Zingiberaceae* is the largest families of the plant kingdom. Its plants tend to be high in medicinal values and provide many useful products for food, spices, medicines, dyes, perfume and esthetics (Jantan et al., 2003; Koga et al., 2016). *Zingiber* is a genus of *Zingiberaceae* with approximately 141 species (Sirirugsa, 1995). *Zingiber zerumbet (L) Smith* is a wild ginger belonging to the *Zingiber* genus and is well-known among local cultures as “Lempoyang,” “Ghatian,” “Yaiimu,” “Jangli adha,” “Awapuhi,” “Zurunbah,” “Hong Qiu Jiang,” and “Hiao Dam.” This particular type of wild ginger grows naturally in damp, shaded parts of the low land and is believed to be native to India and the Malaysian Peninsula (Yob et al., 2011). The traditional uses of ginger are broad, including but not limited to the treatment of nausea, hangovers, migraine headache, morning and motion sickness, worm infestation in children, as well as cuts and bruised skin (Nik Norulaini et al., 2009; Butt and Sultan, 2011; Sahebkar, 2011). Various local groups have been using ginger to provide remedy against allergic diseases including asthma and sinusitis for centuries (Butt and Sultan, 2011; Sahebkar, 2011). As one type of wild ginger, the crude extract as well as the active compounds extracted from the rhizome and leaves of *Z. zerumbet* have been reported to possess various pharmacological properties including anti-inflammatory (Murakami et al., 2002; Jalil et al., 2015), antitumoral (Rashid and Pihie, 2005; Takada et al., 2005; Abdelwahab et al., 2010), antioxidant (Ruslay et al., 2007; Rout et al., 2011), antibacterial (Kumar et al., 2013), antiviral (Epstein-Barr virus) (Murakami et al., 1999), analgesic (Somchit et al., 2005), anti-allergic (Tewtrakul and Subhadhirasakul, 2007) characteristics and usefulness for treating stomach problems (Prakash et al., 2011).

## The Anti-Allergic and Immune Modulation Activities of *Z. zerumbet*

Although there have been quite a number of studies conducted to study the effectiveness of *Z. zerumbet* in a broad range of biological activities related to human health, there has been very few reported studies of *Z. zerumbet* as well as its bioactive compounds focusing on anti-allergy. Increasing levels of allergic diseases, such as allergic rhinitis (AR), atopic dermatitis, asthma and food allergies in many of the developed countries (Carlsen, 2003) are causing significant health problems, especially in children. Therefore, various research has been carried out extensively to combat these diseases (Pawankar et al., 2013). In one of the studies, the ethanolic and aqueous extraction of *Z. zerumbet* were subjected to an *in vitro* investigation for its anti-allergic activities (Tewtrakul and Subhadhirasakul, 2007). This study has shown that the ethanolic and aqueous extracts of *Z. zerumbet* (10–100 μg/mL) inhibited the release of β-hexosaminidase from RBL-2H3 cells as much as 8.4–53.7% (IC_50_ = 91 μg/mL) and 10.9–59.1% (IC_50_ = 68.2 μg/mL), respectively. Several patents were filed due to the exceptional anti-allergic activities shown by *Z. zerumbet*. Among which, a patent by Chaung et al. (2009) provides a method of preparing polar solvent extraction from the root of *Z. zerumbet* as well as the use of this formulation to prevent or to treat an allergic disorder. Another patent by Lin et al. (2013) provides a method of preparing solvent extraction by using ethanol, water, or a mixture of both from the root of *Z. zerumbet* for treating AR or allergic eczema.

The essential oils from rhizomes of *Z. zerumbet* have also been shown to contain several beneficial effects such as analgesic activity (Sulaiman et al., 2010), anti-nociceptive activity (Khalid et al., 2011), and anti-microbial activity (Kader et al., 2010). However, anti-allergic activities involving the essential oils extracted from the rhizomes of *Z. zerumbet* are still yet to be well-reported. Hence, this mini-review focuses on the major bioactive compounds found in the essential oils extracted from the rhizomes of *Z. zerumbet* which have been reported to possess anti-allergic and immunomodulatory properties in order to improve the understanding on the use of *Z. zerumbet* and its bioactive compounds in the treatment of allergy and allergic-related diseases.

## The Anti-Allergic and Immunomodulatory Activities of the Major Bioactive Compounds in the Essential Oils Extracted From the Rhizomes of *Z. zerumbet*

**FIGURE 1:**
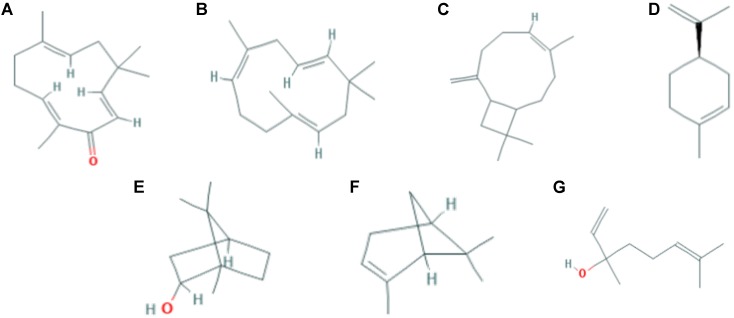
The chemical structure of some of the major bioactive compounds that can be found in *Zingiber zerumbet*. **(A)** Zerumbone **(B)** α-Humulene **(C)** trans-Caryophyllene **(D)** Limonene **(E)** Borneol **(F)** α-Pinene **(G)** Linalool.

Currently, there are only a few zingiber genus that have been reported to contain anti-allergic properties, including *Z. officinale*, *Z. cassumunar*, *Z. zerumbet*, and *Z. mioga* (Tewtrakul and Subhadhirasakul, 2007; Shin et al., 2015). The major bioactive compounds which can be found in the essential oil of *Z. officinale* are α-zingiberene (17.4–32.2%), β-sesquiphellandrene (6.6–27.16%), and geranial (25.9%); for *Z. cassumunar* is sabinene (36.71–53.50%); for *Z. zerumbet* are zerumbone (35.5–84.8%) and pinene (10.3% to 31.4%); for *Z. mioga* is β-phellandrene (26.60%) (Kurobayashi et al., 1991; Sharifi-Rad et al., 2017). Interestingly, zerumbone was found to be exclusively and abundantly present (>80%) in the essential oil extracted from the rhizomes of *Z. zerumbet*, in comparison to other major bioactive compounds extracted from *Z. officinale*, *Z. cassumunar* and *Z. mioga*. According to Shieh et al. (2015), the anti-allergic effects of *Z. zerumbet* may be due to zerumbone as this compound has been shown to effectively inhibit asthma in mice. Apart from zerumbone (35.5–84.8%), the other major compounds that can be found in the essential oils extracted from the rhizomes of *Z. zerumbet* are pinene (10.3–31.4%), humulene (10.03–17.23%), linalool (7.7–17.1%), caryophyllene (6.9–10.2%), borneol (4.78%), and limonene (0.8–1.3%) (**Figure 1**). Among which, it is interesting to note that limonene can only be found in *Z. zerumbet* but not the other *Zingerber* genus (Sun, 2007; Bhuiyan et al., 2008).

The biomedical applications of some of these major bioactive compounds found in *Z. zerumbet* have been previously summarized in several review papers (Calderón-Montaño et al., 2011; Singh et al., 2012; Kalantari et al., 2017). However, the anti-allergic properties of these major bioactive compounds were not included. In this review, the major bioactive compounds found in the essential oils extracted from the rhizomes of *Z. zerumbet*, such as zerumbone, limonene, borneol, pinene, linalool, humulene, and caryophyllene, and their reported anti-allergic and immune modulation activities are summarized in **Table 1**.

**Table 1:** The major bioactive compounds found in *Z. zerumbet* and their reported anti-allergic and immune modulation properties.

Compound	Experimental model	Anti-allergic or immune modulation activities	Concentrations/ Doses of compound used	Mode of application	End-point assessment	Extraction method	Relative quantities in the essential oil extracted from the rhizome of *Z. zerumbet*
Zerumbone	*In vivo* Fournial et al., 2013; Shieh et al., 2015)	Exhibited anti-asthmatic activities in BALB/c mice by decreasing the severity of airway hyperresponsiveness, cytokine secretions and inflammatory cells infiltration.	0.1 – 10 mg/kg Oral route	Co-treatment (zerumbone administration: day 23–39) (OVA challenge: day 28, 35–36, 37–39)	Day 40	Solvent extraction (ethanol, dichloromethane), Supercritical CO_2_, Hydro-distillation	35.5–84.8%
		New topical use for the treatment of cutaneous rednesses.	No data	No data	No data		
Pinene	*In vivo* Nam et al., 2014	Exhibited anti-allergic activities by reducing infiltration of inflammatory cells, IgE level and release of allergic mediators in allergic rhinitis (AR)-induced BALB/c mice.	0.1 – 10 mg/kg Oral route	Pre-treatment (1 h before OVA challenge from day 15–24) Post-treatment (1 h after OVA challenge from day 15–24)	Day 24	Solvent extraction (petroleum ether, pentene and benzene), Hydro-distillation	10.3–31.4%
	*In vitro* Nam et al., 2014	Inhibited activation of NF-κB translocation and mRNA expression of protein mediators in PMACI-induced activation of HMC-1 cells.	0.1 – 10 μg/mL	Pre-treatment (1 h before PMACI challenge)	8 h after challenge		
Humulene	*In vivo* Rogerio et al., 2009	Exhibited anti-asthmatic activities by reducing eosinophil recruitment into the airways of BALB/c mice induced with allergic inflammation.	50 mg/kg Oral route	Pre-treatment (1 h before OVA challenge from day 18–22)	Day 22	Solvent extraction (petroleum ether, pentene and benzene)	10.03–17.23%
Linalool	*In vivo* Mitoshi et al., 2014	Demonstrated protective effects against DNP-human serum albumin- induced passive cutaneous anaphylaxis reaction in ICR mice.	100 mg/kg, Oral route	Pre-treatment (2 h before DNP-HSA challenge)	30 min after challenge	Solvent extraction (petroleum ether, pentene and benzene), Hydro-distillation	7.7–17.1%
	*In vitro* Mitoshi et al., 2014	Exhibited anti-allergic properties in RBL-2H3 cells by reducing levels of mediators’ release.	100 μg/mL	Co-treatment (30 min with calcium ionophore challenge)	30 min after challenge		
Caryophyllene	*In vivo* Passos et al., 2007; Jin et al., 2011	Exhibited anti-allergic activities in OVA-evoked allergic pleurisy in Wistar rats by reducing eosinophil migration, cyclooxygenase (COX) activity and levels of mediators’ release. Exhibited anti-allergic activities in picryl chloride-induced delayed hypersensitivity in ICR mice.	600 mg/kg, Oral route 50 - 300 mg/kg Oral route	Pre-treatment (1 h before bee venom challenge) Post-treatment (24 h after picryl chloride or acetone challenge)	6 h after challenge 25 h after challenge	Solvent extraction (petroleum ether, pentene and benzene), Hydro-distillation	6.9–10.2%
	*In vitro* Jin et al., 2011	Exhibited anti-allergic properties in rat basophilic leukemia (RBL)-1 cells by reducing 5-lipoxygenase (LOX) inhibitory activity as well as levels of mediators’ release.	30 – 300 μg/mL	Pre-treatment (10 min before calcium ionophore challenge)	10 min after challenge		
Borneol	*In vivo* Watanabe et al., 1994	Exerted inhibitory effects on histamine release from abdominal mast cells induced by ovalbumin (OVA).	No data	No data	NA	Solvent extraction (petroleum ether, pentene and benzene)	4.78%
Limonene	*In vivo* Hirota et al., 2012	Reduced *Dermatophagoides farinae*-induced airway remodeling and airway hyperresponsiveness (AHR) in BALB/c mice.	1 mg/kg Intranasal	Pre-treatment (1 h before OVA challenge from day 27–29)	Day 30	Hydro-distillation	0.8–1.3%

### Zerumbone

Zerumbone is a sesquiterpene compound abundantly present (35.5–84.8%) in essential oils extracted from the rhizomes of *Z. zerumbet* (Tewtrakul and Subhadhirasakul, 2007; Sharifi-Rad et al., 2017). A study done by Shieh et al. (2015) showed that zerumbone isolated from *Z. zerumbet* decreased the severity of airway hyperresponsiveness and the accumulation of eosinophils in bronchoalveolar lavage fluid (BALF) collected from OVA-challenge female BALB/c mice. The oral administration of zerumbone (0.1, 1, and 10 mg/kg) also significantly reduced serum anti-OVA IgE levels in mice (Shieh et al., 2015), which further resulted in the reduction of OVA-induced cytokine secretions (IL-4, IL-5, IL-10, and IL-13) in the BALF collected (Shieh et al., 2015). Thus, the authors speculated that zerumbone may have an anti-allergic effect on allergic asthma by suppressing Th2-related cytokines (IL-4, IL-5, IL-10, and IL-13) secretion and consequently reducing IgE production by B cells (Shieh et al., 2015). The data reported in this study was the first known report to provide a rationale for extensive preclinical studies on zerumbone in IgE-mediated allergic asthma.

### Pinene

Pinene is a monoterpene compound that can be isolated from *Z. zerumbet* (Koga et al., 2016) in relatively higher quantities (10.3–31.4%) than other plants from the same genus. The percentages of pinene found in *Z. corallinum* and *Z. cassumunar* were only 2.16–3.23% and 5.2–7.25%, respectively (Koga et al., 2016). In addition, pinene has been reported to attenuate OVA-induced AR in female BALB/c mice by decreasing the infiltration of eosinophils and mast cells in AR nasal mucosa tissue, as well as reducing the level of TNF-α and number of nose rubs in mice orally pre-treated with α-pinene (0.1, 1, or 10 mg/kg) (Nam et al., 2014). The authors even demonstrated that post-treatment of α-pinene in the OVA-induced mice significantly decreased nasal mucosa IgE level and the number of nose rubs (Nam et al., 2014). The *in vitro* study also reported that α-pinene (0.1, 1, or 10 μg/mL) inhibits the production and mRNA expression of TNF-α in PMACI-induced activation of HMC-1 cells (Nam et al., 2014). In term of regulatory mechanism of α-pinene on allergic inflammation, this compound inhibits PMACI-induced activation of NF-κB and IKK-β in HMC-1 cells (Nam et al., 2014). In conclusion, that study suggested that α-pinene was able to exert its anti-allergic effects by interfering the NF-κB/IκB signaling pathway as this pathway is closely related with the inhibition of allergic inflammation in human mast cells (Singh et al., 2011).

### Humulene

Similar to zerumbone, humulene is a sesquiterpene compound that can be found abundantly (10.03–17.23%) in *Z. zerumbet* (Baby et al., 2009). Its key enzyme, α-humulene synthase, has been shown to play a part in the synthesis of zerumbone (Baby et al., 2009). However, other *Zingiber* species such as *Z. nimmonii* and *Z. cassumunar* have been shown to contain higher levels of humulene (19.6–27.7% and 23.92%, respectively), in comparison to *Z. zerumbet*. Rogerio et al. (2009) reported on the inhibitory effect of α-humulene on OVA-induced airway allergic inflammation in female BALB/c mice. They showed that therapeutic treatment with α-humulene (50 mg/kg) may be able to decrease leukocyte recruitment (neutrophils, eosinophils and mononuclear) as well as allergic associated mediators including leukotriene (LT)B_4_ and IL-5 levels in the BALF (Rogerio et al., 2009). The immunohistochemistry staining in this study also revealed the inhibitory effects of α-humulene on the phosphorylation of p65 NF-kB and c-Jun AP-1 subunits, which are the two important modulators for the control production of the Th2 cytokine, IL-5 and the recruitment of leukocytes (Rogerio et al., 2009). These results suggest the potential of α-humulene as a candidate for the treatment of asthma and other allergic diseases.

### Linalool

Linalool is a monoterpene in *Z. zerumbet* that contributes to the aromatic scent of this plant (7.7–17.1%) (Baby et al., 2009). In one study, linalool and other 20 types of natural compounds were shown to inhibit β-hexosaminidase release at the concentration of 100 μg/mL in RBL-2H3 cells induced with calcium ionophore, A23187 (Mitoshi et al., 2014). The study also demonstrated the protective effects of orally administered linalool (100 mg/kg) against DNP-HSA induced passive cutaneous anaphylaxis (PCA) reaction in mice whereby linalool significantly reduced the amount of Evans blue dye present in the exudates collected from ear samples (Mitoshi et al., 2014). In the discussion it was hypothesized that the anti-allergic effects of linalool may be at least in part dependent on the inhibition of NF-κB activation (Mitoshi et al., 2014). However, further investigation should be carried out, as its underlying molecular mechanism remains unelucidated.

### Caryophyllene

Caryophyllene, one of the natural bicyclic sesquiterpenes in *Z. zerumbet*, contributes to the spiciness taste of this plant (Baby et al., 2009). In comparison to *Z. nimmonii* and *Z. officinale* which have been reported to possess 26.9–42.2% and 15.29% of caryophyllene, respectively, *Z. zerumbet* has a relative lower quantity of caryophyllene (6.9–10.2%). Studies have shown that the oral administration of caryophyllene significantly inhibited the oedematogenic response caused by *Apis mellifera* venom in the OVA-sensitized male Wistar rat paws (Passos et al., 2007). Furthermore, the administration of caryophyllene significantly reduced the eosinophil migration at the site of venom induction, leading to reduced levels of tumor necrosis factor alpha (TNF-α), prostaglandin E2 (PGE_2_) and COX activity (Passos et al., 2007). These results strongly suggest the potential of caryophyllene in the treatment of allergic conditions.

Another study conducted by Jin et al. (2011) demonstrated that preincubation of caryophyllene (100 μM) with rat basophilic leukemia-1 (RBL-1) was able to significantly reduce 5-LOX inhibitory activity as well as the release of cysteinyl LTs (LTC_4_/D_4_/E_4_), and the effect was more prominent in comparison to the other two bioactive compounds of *Z. zerumbet* - limonene and pinene (Jin et al., 2011). Caryophyllene also significantly attenuated the antigen-induced degranulation of β-hexosaminidase and phosphorylation of Lyn molecules in RBL-2H3 cell culture (Jin et al., 2011). Furthermore, the *in vivo* immune modulatory effects of caryophyllene were also demonstrated. Caryophyllene was able to significantly inhibit the picryl chloride-induced delayed type hypersensitivity (DTH) response in mice, when given orally (100–300 mg/kg), as evidenced by decreased measurements in the ear thickness of mice (Jin et al., 2011). These findings concluded that caryophyllene exerts anti-allergic activity against mast cell degranulation and offers immune modulatory effects against DTH.

### Limonene

Limonene is the only major bioactive compound found exclusively in *Z. zerumbet* in the Zingiberaceae family (Koga et al., 2016). Limonene has shown a potent reduction in the airway inflammatory reactions with improving asthma symptoms in *Dermatophagoides farinae*-induced allergic airway inflammation of male BALB/c mice (Hirota et al., 2012). One study demonstrated a lowered level of serum total IgE, allergen specific IgG_1_ and allergic associated mediators (IL-5 and IL-13) in mice after inhalation of limonene (1 mg/kg) (Hirota et al., 2012). Additionally, limonene was able to decrease AHR in mice by suppressing the number of eosinophils found in the collected BALF (Hirota et al., 2012). It also significantly reversed allergen-induced lung histopathological changes in mice by lowering perivascular and peribronchial infiltration of eosinophils, goblet cells hyperplasia, airway fibrosis and smooth muscle thickness (Hirota et al., 2012). These findings have shown that limonene may be beneficial as a prophylactic and therapeutic agent for asthma in the future.

## Discussion

Most major bioactive compounds found in the essential oils extracted from the rhizome of *Z. zerumbet* are terpene compounds with long hydrocarbon tails, generally resulting in low polarity (Jiang et al., 2016). As such, a few preferred ways of extracting terpene compounds from *Z. zerumbet* are hydro-distillation and solvent extraction using organic solvents such as ethanol and methanol; or non-polar solvents such as petroleum ether, pentene, hexane, and benzene (Kalantari et al., 2017). A previous study by Tewtrakul and Subhadhirasakul (2007) showed that an ethanolic extract of *Z. zerumbet* containing both polar and non-polar compounds exhibited exceptional anti-allergic effects by inhibiting the release of β-hexosaminidase from RBL-2H3 cells. However, not much attention has been given to the anti-allergic effects of other extracts from *Z. zerumbet* and particularly essential oil, in which the main constituent is zerumbone. Although it has once been reported that the essential oil of *Z. zerumbet* failed to inhibit β-hexosaminidase, the reported yield of essential oil from the whole plant of *Z. zerumbet* in that study was only 3.0%, in comparison to other studies reporting yields ranging from 5 to 13% (Rashid and Pihie, 2005; da Silva et al., 2017). It is also important to note that the essential oils used in that particular study were extracted from the whole plant but not the rhizomes of *Z. zerumbet* alone (Tewtrakul and Subhadhirasakul, 2007). Low levels of bioactive compounds present in the yield in particular terpene compounds may be too low to significantly exhibit an anti-allergic response. Further studies should be carried out with higher yield of extracted essential oil from the rhizomes of *Z. zerumbet*. Distilled hexane can be added during the hydro-distillation process to increase the yield of oil extracted from *Z. zerumbet* (Nik Norulaini et al., 2009). It would be interesting to study whether the essential oils extracted from the rhizomes of *Z. zerumbet* exhibit anti-allergic response as the extracted oil particularly from the rhizomes of *Z. zerumbet* has been proven to have many beneficial properties such as analgesic activity (Sulaiman et al., 2010), anti-nociceptive activity (Khalid et al., 2011) and anti-microbial activity (Kader et al., 2010).

Due to the presence of various bioactive compounds in a plant extract, it is difficult to confirm which bioactive compound contributes to the intended beneficial effects in a disease model (Sasidharan et al., 2011; Katiyar et al., 2012). Therefore, it would be much preferable to identify and isolate the major bioactive compounds present in a plant extract and study them individually. Since many major bioactive compounds found in the essential oil extracted from the rhizomes of *Z. zerumbet* have been shown to be effective in treating allergic responses, it would be important to know whether the doses used in these studies are practical to be translated into clinical studies. The highest oral doses of *Z. zerumbet* extract used in rats and mice were 600 and 300 mg/kg, respectively, equivalent to 97 and 24 mg/kg in humans, according to the human equivalent dose equation (Reagan-Shaw et al., 2016). In terms of bioactive compounds, the highest oral dose of purified zerumbone compound isolated from *Z. zerumbet* used in mice was 10 mg/kg, which is equivalent to 0.81 mg/kg when translated for human consumption. When the translated doses of these bioactive compounds are compared with cromolyn sodium, which is a well-known standard mast cell stabilizer used in many clinical studies to treat various allergic diseases (Burgher et al., 1971; Businco et al., 1983; Burks and Sampson, 1988), the doses used ranging from 8 to 40 mg/kg. Although all of the bioactive compounds found in the essential oil extracted from the rhizomes of *Z. zerumbet* reported in this mini-review are yet to enter clinical trials, this mini-review provides an insight of the recommended doses to be used in any future studies involving these bioactive compounds. Comparisons of the doses used in animal and human studies also indicate the potential of these bioactive compounds to be developed as therapies for the treatment of allergy and allergic-related diseases in future.

## Conclusion

This mini-review summarizes the anti-allergic and immunomodulatory properties of the major bioactive compounds found in the essential oil extracted from the rhizomes of *Z. zerumbet* in order to demonstrate the importance of *Z. zerumbet* in the treatment of allergy and allergic-related diseases, in addition to the other biomedical applications which have been widely reported and extensively studied. Future studies should focus in-depth on exploring the potential therapeutic applications of the major bioactive compounds found in the essential oil extracted from the rhizomes of *Z. zerumbet* toward various allergy-related diseases. It is also important to dissect the mechanism of action of these major bioactive compounds in order to determine how they exert their anti-allergic properties.

## Author Contributions

JT and CT prepared the manuscript. DI reviewed the drafts and provided important information for the completion of this manuscript. CT conceived the idea, reviewed the drafts, and provided important information for the completion of this manuscript.

## Conflict of Interest Statement

The authors declare that the research was conducted in the absence of any commercial or financial relationships that could be construed as a potential conflict of interest.
